# Health dividends of urbanization and spatial spillovers: evidence from urban agglomerations in the Yangtze River Economic Belt

**DOI:** 10.3389/fpubh.2026.1857269

**Published:** 2026-07-01

**Authors:** Peng Sun, Shichang Lu

**Affiliations:** 1 School of Business Administration, Liaoning Technical University, Huludao, China; 2 School of Finance and Trade, Zhuhai College of Science and Technology, Zhuhai, China

**Keywords:** China, environmental governance, health equity, public health, spatial spillover, urbanization

## Abstract

Urbanization serves as a pivotal driver of socio-economic development, yet its impact on public health remains complex and often ambiguous, particularly within ecologically fragile zones such as the Yangtze River Economic Belt (YREB). This study employs a comprehensive econometric framework utilizing panel data from 108 prefecture-level cities in the YREB spanning 2010–2022. We integrate the Spatial Durbin Model (SDM), panel quantile regression, and causal mediation analysis to investigate the direct and indirect impacts of urbanization on public health, proxied by the Crude Death Rate (CDR). The findings reveal a significant inhibitory effect of urbanization on mortality rates, confirming the “urban health dividend.” A notable spatial spillover effect was identified, wherein urbanization in neighboring regions further amplifies local health benefits. Heterogeneity analysis demonstrates that the marginal health benefits of urbanization are significantly greater in high-mortality regions compared to low-mortality regions. Furthermore, mediation tests quantify that medical technology upgrading (24.53%), resident income growth (22.18%), and environmental governance effectiveness (18.65%) are the primary transmission channels. This study may provide regional empirical evidence for understanding the health co-benefits of green urbanization and offers policy implications for advancing coordinated regional development strategies.

## 1 Introduction

Urbanization stands as the quintessential engine of modern socio-economic transformation, fundamentally reshaping global economic landscapes and human settlement patterns while profoundly altering the natural environments upon which public health depends (1–4). The United Nations projects a dramatic surge in the global urban population share, rising from 55% in 2018 to an anticipated 68% by 2050, with Asia and Africa accounting for nearly 90% of this growth (5). In China, the trajectory has been even more compressed and intense: over four decades of reform and opening-up have witnessed the permanent urban resident rate skyrocket from 17.9% in 1978 to 66.2% in 2023—a phenomenon often termed the “Chinese Miracle” involving the migration of hundreds of millions of people (6). However, this rapid urbanization is not merely a demographic shift; it represents a critical juncture where the future of public health hangs in the balance. We are currently facing an urgent reality characterized by a “double-edged sword” effect: while urbanization offers unprecedented opportunities for health improvement, it simultaneously exacerbates complex health risks that threaten to undermine these gains.

The urgency of this challenge is underscored by the divergent impacts of urbanization on public health. On one hand, the “urban health dividend” hypothesis posits that cities act as crucibles for health improvement, enhancing access to medical resources, upgrading sanitation infrastructure, and facilitating the diffusion of health knowledge through agglomeration effects (7–9). This view suggests that urbanization reduces infant mortality and infectious disease incidence by raising income levels and expanding public service coverage (10). Conversely, the “urban health penalty” hypothesis warns that disordered expansion can trigger environmental degradation, increase exposure to occupational hazards, and intensify lifestyle-related non-communicable diseases, thereby offsetting or even reversing health dividends (2, 3, 11). In the post-pandemic era, this tension has become even more acute. Cross-regional transmission of infectious diseases, coupled with the rising burden of chronic conditions driven by sedentary lifestyles and pollution, necessitates an immediate re-evaluation of how urbanization shapes health outcomes. The existing literature, however, remains fragmented and often fails to capture the dynamic, spatially interdependent nature of these challenges.

Existing empirical studies on the relationship between urbanization and health are extensive yet inconclusive, largely due to methodological limitations that obscure the true complexity of the issue. Early research, predominantly based on cross-sectional data, tended to support a linear positive relationship, arguing that urbanization uniformly improves health metrics (9, 10). However, as research scales have refined and time series extended, scholars have increasingly identified significant heterogeneity and context-dependency. For instance, Ezzati et al. (12) found that in sub-Saharan Africa, rapid urbanization exacerbated health inequalities due to slum proliferation rather than reducing mortality. Similarly, within the Chinese context, while Zhang et al. (4) confirmed a positive effect of urbanization on life expectancy He et al. (7) and Liang et al. (10) demonstrated that in regions with weak environmental regulation, urbanization imposed a significant negative impact on respiratory disease mortality. These divergent conclusions stem primarily from three critical gaps in current scholarship: First, most studies rely on static panel models, neglecting the spatiotemporal dynamic dependencies inherent in urbanization—i.e., the health outcomes of one city are intrinsically linked to the urbanization levels of neighboring regions (13–15). Second, existing literature predominantly focuses on mean regression, obscuring differential impacts across regions with varying health baselines (e.g., high-mortality lagging areas vs. low-mortality developed areas) and lacking attention to tail characteristics (16). Finally, discussions on mediating mechanisms often remain at the level of theoretical deduction or single-channel testing, failing to systematically deconstruct multi-dimensional pathways such as “medical technology upgrading,” “resident income growth,” and “environmental governance effectiveness” (17).

These limitations are particularly salient in the context of the Yangtze River Economic Belt (YREB), a national strategic corridor spanning China's eastern, central, and western gradients. Covering 11 provinces and 108 prefecture-level cities, the YREB contributes over 45% of China's GDP and hosts 40% of its population, yet it exhibits stark developmental fractures and ecological fragility. While the downstream Yangtze River Delta has entered a post-industrialization stage, parts of the middle and upper reaches still face monolithic industrial structures, severe environmental pollution, and scarce medical resources (18). Crucially, the YREB bears the dual mission of “jointly promoting great protection without major development” and “high-quality development.” Its urbanization model is shifting from scale expansion to quality enhancement, offering an ideal policy scenario to test whether green urbanization can achieve health co-benefits (19). However, existing research on this region has largely focused on economic growth, technological innovation, or single environmental indicators, with few studies systematically evaluating the net effect of urbanization on public health and its spatial transmission mechanisms (4, 20). Particularly in the post-pandemic era, the importance of cross-regional joint prevention and control of health risks has become increasingly prominent, necessitating a reconstruction of the theoretical framework for urbanization and health governance from a regional collaborative perspective (21).

In light of this urgent reality and the identified research gaps, this study utilizes panel data from 108 prefecture-level cities in the YREB spanning 2010–2022. We construct a comprehensive econometric framework integrating the Spatial Durbin Model (SDM), quantile regression, and causal mediation analysis to explore the complex, spatially dependent impacts of urbanization on public health. Our approach moves beyond static averages to reveal how urbanization affects regions with different health baselines and identifies the precise channels through which health benefits are transmitted. The marginal contributions of this study are fourfold: First, we confirm a significant inhibitory effect of urbanization on mortality rates at the city level within the YREB and identify a spatial disequilibrium characterized by a “weak East, strong West” pattern. Second, transcending the limitations of traditional spatial econometric models, we employ quantile regression to reveal that urbanization yields greater marginal health benefits in regions with high mortality rates, indicating its role as a vital equalizer in narrowing regional health disparities. Third, by introducing a triple mediation mechanism test, we quantify the relative importance of medical technology upgrading, resident income growth, and environmental governance efficacy, offering policymakers precise targets for intervention. Fourth, by grounding the analysis in the unique institutional context and ecological constraints of the YREB, this study provides a “Chinese solution” for achieving the UN Sustainable Development Goals (SDG 3: Good Health and Wellbeing; SDG 11: Sustainable Cities and Communities) in regions undergoing rapid urbanization globally. Through this investigation, we aim to provide actionable insights for optimizing urbanization strategies to maximize public health dividends while mitigating associated risks.

## 2 Study area and data sources

### 2.1 Regional overview

This study focuses on 108 prefecture-level and above cities within the Yangtze River Economic Belt (YREB). Spanning China's eastern, central, and western geographical gradients, the region encompasses core national strategic zones, including the Yangtze River Delta urban agglomeration, the middle reaches of the Yangtze River urban agglomeration, and the Chengdu-Chongqing twin-city economic circle. It is characterized by high spatial heterogeneity and policy representativeness. As the development axis with the highest economic density, population concentration, and most critical ecological functions in China, the YREB contributes approximately 45% of the national GDP and over 40% of the total population. Moreover, it bears the dual mission of “prioritizing ecological conservation over large-scale development” and pursuing “high-quality development.” Consequently, the trajectory of its urbanization process and the evolution of public health serve as a paradigmatic case for understanding the mechanisms of coordinated regional development in China. Due to limitations in the completeness of statistical data for certain county-level cities or remote areas (e.g., missing early mortality registrations and inconsistent urbanization rate metrics), this study ultimately screened for a panel sample of 108 cities characterized by strong data continuity and high indicator comparability to ensure the robustness and reliability of the empirical analysis. Although the sample does not cover all administrative units across the entire basin, the selected cities spatially reproduce the core pattern of the YREB, characterized by “dense distribution in the east, sparse distribution in the west, and agglomeration along the river.” The sample demonstrates sufficient representativeness in terms of economic development levels, public service capabilities, and ecological environmental pressure, effectively supporting both macro-trend inference and micro-mechanism identification.

### 2.2 Data sources

The primary data for this study were compiled from the China City Statistical Yearbook (2011–2023), statistical communiques issued by provincial and municipal bureaus, and the open database of the National Health Commission of China. Additionally, gridded population Digital Elevation Model (DEM) and health-related derivative data were obtained from the Resource and Environmental Science and Data Center of the Chinese Academy of Sciences. Regarding variable definitions, the core explanatory variable, urbanization rate, is measured by the proportion of the permanent resident population living in urban areas. The dependent variable, public health level, is proxied by the Crude Death Rate (CDR) (deaths per 1,000 population). Although CDR is an inverse indicator, it effectively reflects regional medical supply efficiency and environmental risk exposure, particularly after controlling for age structure. To address data discontinuities and minor missing values arising from administrative division adjustments or changes in statistical standards (e.g., city mergers around 2015 and disruptions caused by the COVID-19 pandemic in 2020), we employed the linear interpolation method for imputation. Finally, descriptive statistics and outlier treatment were conducted for all continuous variables prior to regression analysis.

## 3 Mechanism analysis and hypotheses

**Figure 1:**
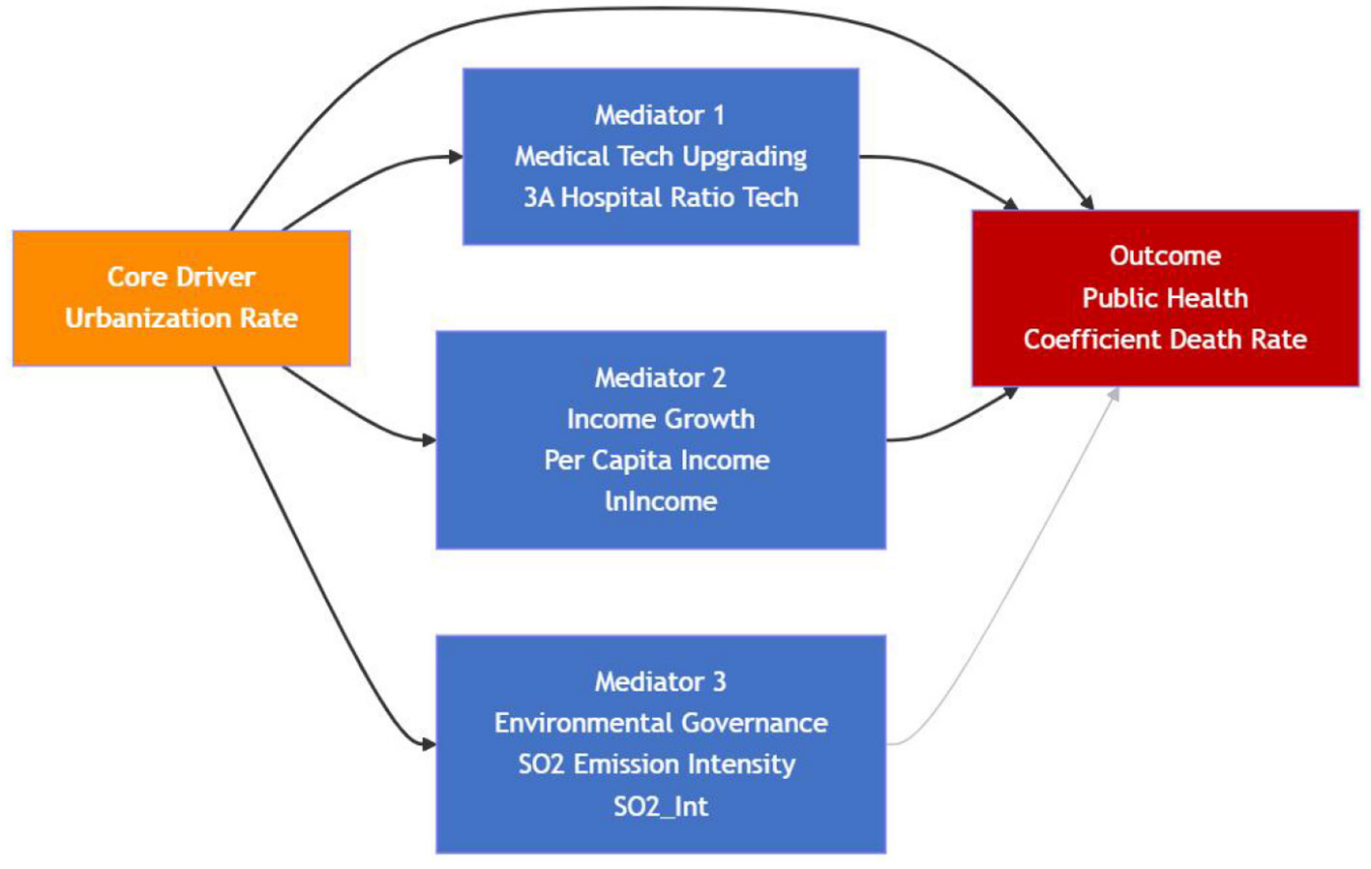
Theoretical mechanism diagram.

As the core driver of modern social structural transformation, the relationship between urbanization and public health is not a simple linear correspondence but a complex dynamic system involving resource agglomeration, economic distribution, and ecological feedback. Based on the dual hypotheses of “urban health dividend” and “urban penalty,” and considering the specific context of the YREB, this paper constructs a theoretical analysis framework from the dimensions of direct effects and indirect transmission pathways (Figure 1).

### 3.1 Direct impact of urbanization on public health

Through population agglomeration and spatial restructuring, the urbanization process exerts a direct and profound impact on public health. According to classical urban epidemiological theory, this impact exhibits a significant “double-edged sword” characteristic (3). On one hand, the “urban health dividend” hypothesis posits that urbanization is a key engine for improving public health levels. Firstly, economies of scale enable cities to provide high-quality public health infrastructure (e.g., sewage treatment plants, waste disposal systems) and medical service networks at a lower marginal cost, significantly reducing the incidence and mortality of infectious diseases (7, 9). Secondly, the knowledge spillover effect accelerates the dissemination of health literacy; urban residents have easier access to scientific health information and preventive knowledge, thereby improving lifestyles (8). Finally, the agglomeration effect facilitates the concentration of medical talent and rapid technological iteration, enhancing the survival rates of emergency and critical cases (22). In the Chinese context, rapid urbanization is accompanied by a government-led strategy for the equalization of basic public services, which further reinforces these positive effects (1). On the other hand, the “Urban Penalty” hypothesis suggests that rapid urbanization may induce health risks. High-density population agglomeration can accelerate the spread of respiratory infectious diseases (23); environmental pollution caused by industrial emissions and traffic congestion (e.g., PM2.5, SO_2_, noise) directly increases the burden of cardiopulmonary diseases (2, 24). Furthermore, psychological stress, sedentary behavior, and dietary changes triggered by the fast pace of urban life have also increased the incidence of non-communicable chronic diseases (11, 25).

However, for the YREB, which is in a critical period of high-quality development transformation, the negative externalities of urbanization are gradually being curbed with the implementation of the “Joint Conservation and Protection” strategy and the strengthening of environmental regulations. Meanwhile, the positive advantages in resource allocation efficiency are becoming increasingly prominent. Based on this, we propose the core hypothesis:

H1: After controlling for other variables, the increase in urbanization rate has a significant net positive promoting effect on the public health level of the Yangtze River Economic Belt.

### 3.2 Indirect transmission mechanisms of urbanization on public health

Urbanization not only directly affects health outcomes but also exerts indirect impacts by reshaping the distribution of medical resources, altering residents' economic capabilities, and affecting ecological environmental quality. This paper selects medical technology level (Tech), resident income level (lnIncome), and environmental governance effectiveness (SO_2__Int) as mediating variables to deeply analyze the internal transmission mechanisms.

#### 3.2.1 Medical technology upgrading path (Tech)

Urbanization acts as a catalyst for the spatial optimization of medical resources. According to Central Place Theory, high-level medical facilities tend to be located in city centers with dense populations and convenient transportation to maximize service coverage (26). As the urbanization rate increases, the expansion of the urban population creates a huge market for medical demand, attracting high-quality medical capital and top-tier talent, manifested as an increase in the proportion of tertiary hospitals (Tech). A high level of medical technology improves health through the following mechanisms: (1) Enhanced Diagnostic and Treatment Capabilities: Tertiary hospitals possess advanced diagnostic equipment and specialized treatment methods, significantly reducing the mortality rate of difficult and critical illnesses (17). (2) Strengthened Preventive Care: High-level hospitals often undertake regional public health functions, promoting the popularization of preventive services such as vaccination and cancer screening (22). (3) Technology Diffusion Effect: Technological progress in central cities radiates to surrounding areas through medical consortia and telemedicine, improving the overall regional medical level (27). Therefore, urbanization reduces mortality rates by promoting the upgrading of medical technology levels.

H2: Urbanization indirectly promotes public health levels by enhancing the medical technology level (Tech).

#### 3.2.2 Resident income growth path (lnIncome)

Urbanization is the spatial carrier of economic growth and an important source of income growth for residents. Industrial agglomeration and the deepening of division of labor improve labor productivity and create a large number of high value-added employment positions, thereby significantly increasing the per capita disposable income of urban residents (lnIncome) (28). According to the Grossman Health Production Function theory, income is a core element of health investment (29). Income growth improves health through the following channels: (1) Enhanced Payment Ability: Higher income enables residents to afford better medical services, nutritious food, and commercial health insurance, reducing the phenomena of “poverty caused by illness” and “abandoning treatment due to poverty” (1). (2) Optimized Health Behavior: Improved economic conditions encourage residents to increase health investments such as physical exercise and leisure tourism, while reducing high-risk behaviors (2). (3) Improved Living Conditions: Income growth supports residents in moving to communities with better infrastructure and superior environments, indirectly reducing health risks (30). Therefore, urbanization improves public health by raising resident income levels.

H3: Urbanization indirectly promotes public health levels by increasing resident income levels (lnIncome).

#### 3.2.3 Environmental governance optimization path (SO_2__Int)

Traditional views suggest that urbanization exacerbates environmental pollution, but the Environmental Kuznets Curve (EKC) hypothesis posits that once urbanization crosses a certain threshold, industrial structure upgrading and strengthened environmental regulations will drive environmental quality improvement (31). Under the “Ecological Priority” strategy of the YREB, the urbanization process is accompanied by the elimination of highly polluting industries and the introduction of green technologies, manifested as a decline in industrial sulfur dioxide emission intensity (SO_2__Int), i.e., an improvement in environmental governance effectiveness. The improvement of environmental quality is directly related to health: (1) Reduced Exposure Risk: The decline in pollutant concentrations such as sulfur dioxide directly reduces the incidence and mortality risks of respiratory diseases (e.g., asthma, COPD) and cardiovascular diseases (21, 24). (2) Restoration of Ecosystem Services: Environmental governance is often accompanied by increased green space and water restoration, providing spaces for psychological healing and exercise, thereby enhancing residents' physical and mental health (32, 33). (3) Long-term Cumulative Effect: Continuous environmental governance reduces the accumulation of toxic substances in the human body, lowering long-term health risks such as cancer (34). The urbanization reduces mortality rates by compelling the improvement of environmental governance effectiveness.

H4: Urbanization indirectly promotes public health levels by enhancing environmental governance effectiveness (reducing SO_2__Int).

## 4 Variable description and methodology

### 4.1 Variable description

#### 4.1.1 Dependent variable

Public health level is the primary focus of this study. Given the limitations in accessing individual health data at the macro level, and considering that mortality rates serve as a comprehensive, inverse indicator reflecting the efficiency of regional healthcare supply, exposure to environmental risks, and residents' quality of life, we selected the Crude Death Rate (CDR) as the proxy variable for public health. Specifically, this is measured as the number of deaths per 1,000 permanent residents (17, 26, 35). A lower value indicates superior regional public health status. Although the CDR is significantly influenced by age structure, this study incorporates the degree of population aging as a control variable. Furthermore, we employed the age-standardized mortality rate in robustness checks to ensure the unbiasedness of the estimation results.

#### 4.1.2 Core independent variable

The urbanization rate is the key metric for gauging regional urbanization progress (7, 8). This study measures it using the permanent population urbanization rate, defined as the proportion of the urban permanent population to the total population in a region. This indicator reflects not only the spatial agglomeration of population from rural to urban areas but also implies industrial upgrading, lifestyle transitions, and improved coverage of public services (9). According to the Push-Pull Theory, urbanization improves healthcare accessibility through agglomeration economies but may also negatively impact health due to “urban diseases” (e.g., congestion, pollution). Therefore, the net effect requires identification through empirical modeling.

#### 4.1.3 Control variables

To isolate the interference of other factors on public health and accurately identify the net effect of urbanization, we introduced the following five categories of control variables (17, 21, 24, 32, 36): Economic Development Level (lnPGDP): Measured by the Gross Regional Product per capita (per capita GDP), processed with a natural logarithm transformation. Based on the Grossman Health Demand Model, economic growth enhances nutritional levels and healthcare payment capacity by increasing resident income and government fiscal input, thereby improving health status. Medical Resource Stock (Med): proxied by the number of hospital beds per 1,000 people. This indicator directly reflects the hardware supply capacity of regional healthcare services, serving as a fundamental factor determining the success rate of disease treatment. Environmental Pollution Level (Pollution): Measured by industrial wastewater discharge or industrial soot and dust emissions (standardized). Environmental pollution acts as a significant external negative effect threatening public health; high pollution levels often lead to increased incidence of respiratory and cardiovascular diseases. Ecological Endowment (Green): Characterized by the green coverage rate of built-up areas. Green spaces play a crucial role in regulating microclimates, alleviating psychological stress, and promoting physical activity, constituting a vital component of urban ecological health capital. Population Aging Degree (Aging): Measured by the proportion of the population aged 65 and above. Given that the elderly are a high-risk group for mortality, this variable is critical for controlling demographic structural differences and avoiding spurious regression.

#### 4.1.4 Mediating variables

To deeply reveal the internal transmission mechanisms through which urbanization affects public health, we selected mediating variables across three dimensions (4, 13, 20): Medical Technology Level (Tech): Measured by the proportion of Class III Grade A hospitals (the highest tier in China's healthcare system) within the region. These hospitals represent the pinnacle of medical technology. This indicator reflects the agglomeration effect and technological spillover of high-quality medical resources during urbanization, serving as a key technical channel linking urbanization to health outcomes. Resident Income Level (lnIncome): Calculated as the natural logarithm of the per capita disposable income of urban residents. Income is a direct input factor in health production. Urbanization enhances individual payment capacity for health management, nutritional intake, and preventive care by creating employment opportunities and raising incomes. Environmental Governance Effectiveness (SO_2__Int): Measured by industrial sulfur dioxide emission intensity (SO_2_ emissions per unit of GDP). This indicator reflects the capacity for economic growth to decouple from environmental degradation during urbanization. If urbanization is accompanied by industrial structure optimization and green technological progress, it will reduce pollution emissions per unit of output, thereby indirectly improving public health by enhancing environmental quality.

### 4.2 Methodology

This paper aims to empirically examine the impact, spatial characteristics, heterogeneity, and internal transmission mechanisms of the urbanization process on public health in the YREB. To ensure the reliability of conclusions and the rigor of causal inference, we constructed a comprehensive econometric analysis framework. This framework encompasses benchmark regression, spatial econometrics, quantile regression, Instrumental Variable (IV) methods, and mediation effect models.

#### 4.2.1 Baseline regression model

To preliminarily identify the net impact of urbanization on public health, this study initially constructs a Two-Way Fixed Effects model. This specification is instrumental in controlling for time-invariant city-specific heterogeneity (e.g., geographical endowments, historical, and cultural contexts) and time-specific trends common to all cross-sections (e.g., macroeconomic policy shocks, technological progress), thereby mitigating potential omitted variable bias (13, 21). The baseline model is specified as follows Equation 1:


$$\begin{array}{l} Health_{it}=\alpha_{0}+\alpha_{1}Urban_{it}+\underset{k}{\sum }\;\beta_{k}Control_{it}+\mu_{i}+\delta_{t}+\varepsilon_{it} \end{array}$$ (1)

Where *i* denotes the city and *t* represents the year. *Health*_*it*_ is the dependent variable, proxied by the Crude Death Rate (*Death*_*rate*) to measure public health status, where a lower value indicates superior health outcomes. *Urban*_*it*_serves as the core independent variable, defined as the population urbanization rate. *Control*_*it*_ represents a vector of control variables, including the level of economic development (*lnPGDP*), stock of medical resources (*Med*), environmental pollution levels (*Pollution*), ecological endowment (*Green*), and the degree of population aging (*Aging*). μ_*i*_ and δ_*t*_ denote the city-specific fixed effects and year fixed effects, respectively, while ε_*it*_ is the random error term.

#### 4.2.2 Spatial autocorrelation test: Global Moran's I

Given the significant geographical proximity and spatial spillover characteristics of public health, neglecting spatial dependence may lead to biased estimation results (14, 27). Prior to conducting spatial econometric regression, this study employs the Global Moran's I statistic to test for spatial clustering patterns in urban public health across the YREB (15). The calculation formula is as follows Equation 2:


$$\begin{array}{l} I=\frac{n}{\overset{n}{\underset{i=1}{\sum }}\;\overset{n}{\underset{j=1}{\sum }}\;w_{ij}}\cdot \frac{\overset{n}{\underset{i=1}{\sum }}\;\overset{n}{\underset{j=1}{\sum }}\;w_{ij}\left(y_{i}-\bar{y}\right)\left(y_{j}-\bar{y}\right)}{\overset{n}{\underset{i=1}{\sum }}\;{\left(y_{i}-\bar{y}\right)}^{2}} \end{array}$$ (2)

Where *n* denotes the number of city samples, *y*_*i*_ and *y*_*j*_ represent observations of the public health indicator for cities *i* and *j*, respectively, and $\bar{y}$ is the sample mean. *w*_*ij*_ denotes elements of the spatial weight matrix. This study constructs an inverse distance matrix (*W*_*dist*_), defined as *w*_*ij*_ = 1/*d*_*ij*_ (*i*≠*j*), where *d*_*ij*_ is the spherical distance between geographical centroids of two cities.

#### 4.2.3 Spatial Durbin Model (SDM)

This paper employs Wald and LR tests to determine whether the Spatial Durbin Model (SDM) can be simplified to a Spatial Autoregressive Model (SAR) or a Spatial Error Model (SEM). Based on the statistical rigor of these tests, the study ultimately selects a Spatial Durbin Model with two-way fixed effects for the regression analysis. This approach acknowledges that urbanization not only impacts local public health but may also generate spatial spillover effects on neighboring regions through the channels of factor mobility, technology diffusion, and environmental linkages (21). The SDM incorporates spatial lag terms for both the dependent and explanatory variables, enabling unbiased decomposition of direct and indirect effects (33). The model is specified as follows Equation 3:


$$\begin{array}{l} Health_{it}=\rho W\cdot Health_{it}+\alpha_{1}Urban_{it}+\theta W\cdot Urban_{it} \end{array}$$

$$\begin{array}{l} +\underset{k}{\sum }\;\beta_{k}Control_{it}+\underset{k}{\sum }\;\lambda_{k}W\cdot Control_{it}+\mu_{i}+\delta_{t}+\varepsilon_{it} \end{array}$$ (3)

Where *W* is the spatial weight matrix, *W*·*Health*_*it*_ is the spatial lag of the dependent variable, and the coefficient ρ reflects the degree of spatial autocorrelation. *W*·*Urban*_*it*_ and *W*·*Control*_*it*_ represent spatial lags of the core explanatory and control variables, respectively. The coefficients θ and λ_*k*_ capture the influence of neighboring regions' variables on the local area.

#### 4.2.4 Heterogeneity analysis: panel quantile regression

While baseline and spatial regressions primarily focus on the “average” effect of urbanization on public health, they may obscure structural differences across varying health levels (3, 10, 13, 21). To examine heterogeneous impacts of urbanization across different health distributions (e.g., high-mortality vs. low-mortality cities), this study employs a panel quantile regression model. The specification is as follows Equation 4:


$$\begin{array}{l} Q_{\tau }\left(Health_{it}|X_{it}\right)=\alpha_{\tau }+\alpha_{1,\tau }Urban_{it}+\underset{k}{\sum }\;\beta_{k,\tau }Control_{it} \end{array}$$

$$\begin{array}{l} +\mu_{i}+\delta_{t} \end{array}$$ (4)

Where *Q*_τ_(·) denotes the conditional quantile function at quantile point τ (τ∈ {0.1,0.25,0.5,0.75,0.9}). The coefficient α_1, τ_ reflects the marginal effect of urbanization on public health at the τ-th quantile. By comparing coefficient variations across different τ values, this analysis identifies whether urbanization exerts stronger improvement effects on cities with poorer (high-mortality quantiles) or better (low-mortality quantiles) health conditions, thereby testing for potential “convergence” or “divergence” effects (23).

#### 4.2.5 Endogeneity treatment: instrumental variable approach (2SLS)

Although the Spatial Durbin Model (SDM) mitigates omitted variable bias to some extent, the potential bidirectional causality between urbanization and public health—for instance, regions with higher health levels may attract greater population inflows, thereby accelerating urbanization—can still lead to endogenous bias in the estimation results (1, 4, 13, 20). To obtain consistent and unbiased estimators, this study selects the “Relief Degree of Land Surface” (RDLS) as an instrumental variable (IV) for the core explanatory variable, “urbanization rate,” and employs the Two-Stage Least Squares (2SLS) method for causal identification (28). The Equations 5, 6 are as follows:


$$\begin{array}{l} Urban_{it}=\gamma_{0}+\gamma_{1}IV_{it}+\underset{k}{\sum }\;\gamma_{k}Control_{it}+\mu_{i}+\delta_{t}+u_{it} \end{array}$$ (5)

$$\begin{array}{l} Health_{it}=\alpha_{0}+\alpha_{1}{\hat{Urban}}_{it}+\underset{k}{\sum }\;\beta_{k}Control_{it}+\mu_{i}+\delta_{t}+v_{it} \end{array}$$ (6)

Where $\hat{Urban_{it}}$ represents fitted values from the first-stage regression. If the first-stage F-statistic exceeds 10 (rejecting the weak instrument hypothesis) and α_1_ remains significant in the second stage, this confirms the causal effect of urbanization on public health improvement.

#### 4.2.6 Mechanism test: mediation effect model

To dissect the intrinsic transmission pathways through which urbanization affects public health, this study constructs a mediation effect model based on the three-dimensional framework of “Medical Technology Upgrading—Resident Income Growth—Environmental Quality Improvement” (17). Using stepwise regression, the following Equations 7, 8and9 are specified:


$$\begin{array}{lll} Health_{it} & = & c\cdot Urban_{it}+\sum \beta_{k}Control_{it}+\mu_{i}+\delta_{t}+\varepsilon_{1it} \end{array}$$ (7)

$$\begin{array}{lll} M_{it} & = & a\cdot Urban_{it}+\sum \lambda_{k}Control_{it}+\mu_{i}+\delta_{t}+\varepsilon_{2it} \end{array}$$ (8)

$$Health_{it}\;=c^{'}\;\cdot Urban_{it}+b\cdot M_{it}+\sum \varphi_{k}Control_{it}+\mu_{i}+\delta_{t}+\varepsilon_{3it}$$ (9)

Where *M*_*it*_ denotes the mediating variable, corresponding respectively to: medical technology level (*Tech*, proportion of Grade-A tertiary hospitals); resident income level (*lnIncome*, per capita disposable income of urban residents); and environmental governance performance (*SO*_2__*Int*, industrial sulfur dioxide emission intensity).

## 5 Results and discussion

### 5.1 Spatiotemporal characteristics of public health

**Figure 2:**
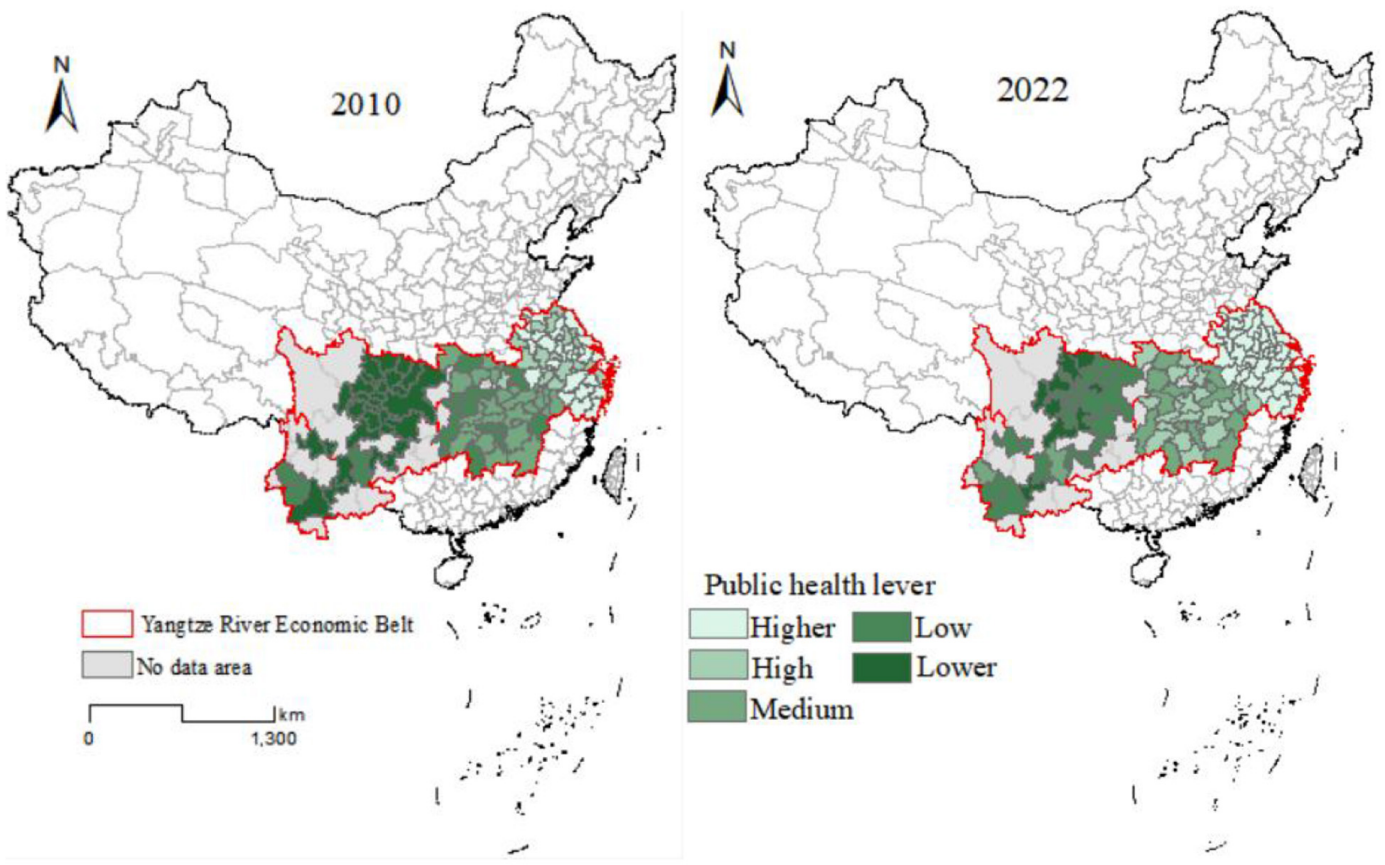
Spatial distribution of public health level.

Using ArcGIS 10.8, this study classifies the spatiotemporal characteristics of public health in the YREB into five categories based on the natural breaks method (Figure 2). From 2010 to 2022, the public health level in the region (measured by mortality rate) exhibited significant temporal evolution. Specifically, data from 2010 revealed pronounced spatial heterogeneity in public health conditions, with relatively higher levels in the eastern coastal areas and lower levels in the central and western regions. In that year, cities along the eastern coast, such as Shanghai and Jiangsu, reported mortality rates in the lower range, indicating superior public health conditions. In contrast, cities in central and western regions, such as Sichuan and Chongqing, exhibited higher mortality rates, ranging from 6.223 to 7.412. By 2022, however, there was a general improvement in public health levels across the belt, with a widespread decline in mortality rates. Despite this progress, disparities between the east and west persist, although they have narrowed compared to 2010. This suggests that over the past decade, the implementation of regional coordinated development strategies and the optimization of medical resource allocation have led to varying improvements in public health across cities in the YREB.

Spatially, public health levels in the region demonstrate distinct clustering and gradation characteristics. First, cities with high public health levels are primarily concentrated in the eastern coastal zone, forming a relatively contiguous high-value cluster. This is likely attributable to the region's higher economic development, abundant medical resources, and superior living standards, all of which contribute to favorable public health outcomes. Second, cities with moderate public health levels are predominantly located in central provinces, such as Hubei and Hunan, serving as a transitional zone between the east and west. Finally, cities with low public health levels are mostly found in the western inland regions, particularly in the mountainous southwest, where poor natural conditions, weak economic foundations, and limited access to medical services have resulted in persistently low public health levels. Notably, while the entire region showed improvements by 2022, the western region's progress was relatively modest, and the absolute gap with the eastern region remains substantial.

### 5.2 Baseline regression analysis

To empirically examine the impact of the urbanization process on public health levels and its underlying mechanisms, this study conducts a baseline regression analysis based on panel data from 108 prefecture-level cities in the YREB from 2010 to 2022, using a Two-way Fixed Effects Model. By controlling for individual heterogeneity and time trends, we progressively introduce a series of control variables, namely economic development, medical resources, environmental pollution, ecological construction, and population structure, to identify the net effect of the urbanization rate (Urban) on the population death rate (Death_rate). Table 1 reports the specific regression results.

**Table 1:** Benchmark regression results of urbanization on public health.

Variables	(1)	(2)	(3)	(4)
*Urban*	−0.038^***^	−0.032^***^	−0.026^***^	−0.024^***^
	(0.003)	(0.003)	(0.002)	(0.002)
*lnPGDP*		−0.015^***^	−0.012^***^	−0.010^***^
		(0.002)	(0.002)	(0.002)
*Med*			−0.003^**^	−0.002^*^
			(0.001)	(0.001)
*Pollution*				0.015^***^
				(0.003)
*Green*				−0.004^**^
				(0.002)
*Aging*				0.028^***^
				(0.003)
*City/Year Fe*	Yes	Yes	Yes	Yes
*_cons*	7.852^***^	8.124^***^	7.956^***^	7.685^***^
	(0.152)	(0.168)	(0.145)	(0.138)
*N*	1,404	1,404	1,404	1,404
*R* ^2^	0.412	0.586	0.673	0.754
*R* ^2^ *_a*	0.398	0.574	0.662	0.745

The parentheses indicate robust standard errors; ^***^, ^**^, and ^*^, respectively indicate significance at the 1%, 5%, and 10% levels.

Column (4) of Table 1 presents the full model regression results incorporating all control variables and serves as the core basis for the baseline analysis in this paper. The results show that the regression coefficient of the urbanization rate (Urban) is −0.024 and highly significant at the 1% statistical level. This indicates that, after controlling for per capita GDP, medical resources, environmental pollution, green space area, and the degree of aging, and absorbing city-specific fixed effects and year fixed effects, the urbanization process has a significant promoting effect on reducing the population death rate. Specifically, for every 1 percentage point increase in the urbanization rate, the urban population death rate decreases on average by 0.024 per thousand. This negative effect demonstrates the applicability of the “urbanization health dividend” in the YREB, which may be attributed to several factors. First, urbanization brings about the agglomeration effect of public resources. As the population concentrates in cities, high-quality medical facilities, public health service systems, and emergency networks can cover more people more efficiently, thereby reducing death risks caused by inadequate access to healthcare. Second, urbanization is accompanied by the improvement of infrastructure, such as the popularization of clean drinking water, sewage treatment systems, and improved sanitary living environments, which directly block the transmission routes of infectious diseases. Finally, although the urban lifestyle may bring negative factors such as sedentary behavior, it generally raises residents' health awareness and literacy, making chronic disease management and preventive healthcare more widespread.

It is worth noting that as control variables are gradually added (from Column 1 to Column 4), the absolute value of the Urban coefficient converges from 0.038 to 0.024, while remaining significant at the 1% level. This suggests that part of the health improvement effect brought by urbanization is indirectly achieved through channels such as economic growth, increased medical investment, and environmental governance. For example, after adding per capita GDP in Column (2), the coefficient decreases, indicating that part of the health dividend of urbanization is realized by promoting economic development, increasing residents' income levels, and thereby improving nutritional status and living conditions.

In addition to the core explanatory variables, the regression results of other control variables are consistent with theoretical expectations and economic reality, further verifying the rationality of the model specification. The coefficient of the economic development level (lnPGDP) is −0.010 and significant at the 1% level. This corroborates the basic logic of the Preston curve, namely that economic growth can extend life expectancy by increasing public health expenditure, improving the living environment, and enhancing nutritional levels. The coefficient of medical resources (Med) is −0.002, significantly negative at the 10% level. Although the absolute value of the coefficient is small, considering the large base number of beds, its marginal contribution is still considerable. This reflects the direct “safety net” role of improving medical service supply capacity in reducing the mortality rate of critically ill patients. The coefficient of environmental pollution (Pollution) is 0.015 and significantly positive at the 1% level. This result strongly confirms the damaging effect of environmental pollution on public health. Some midstream cities in the YREB are still in the mid-stage of industrialization, with a high proportion of heavy and chemical industries. Pollutant emissions significantly increase the incidence of respiratory and cardiovascular diseases, thereby driving up the overall mortality rate. This also inversely illustrates the health benefits of environmental governance measures in the recent “Great Protection of the Yangtze River” strategy. The coefficient of ecological construction (Green) is −0.004, significantly negative at the 5% level. This indicates that an increase in per capita park green space area helps reduce the mortality rate, reflecting the positive health externalities of urban green spaces in alleviating mental stress, promoting physical exercise, and regulating the microclimate (e.g., alleviating the heat island effect). The coefficient of population aging (Aging) is 0.028, significantly positive at the 1% level. This is a direct manifestation of demographic laws, as the elderly population, due to physical function decline and high incidence of chronic diseases, is the main bearer of death risks.

### 5.3 Global spatial autocorrelation analysis

For public health levels, due to population mobility, cross-regional transmission of environmental pollution, diffusion of medical technology, and coordination of economic policies between regions, the health levels of cities are often not isolated but show significant spatial dependence. If this spatial correlation is ignored and OLS is directly used for estimation, it may lead to biased or invalid parameter estimation. Therefore, before conducting spatial econometric modeling, it is necessary to first test whether there is spatial autocorrelation in the public health levels of cities in the YREB. This study uses the Global Moran's I as the measurement index to test the spatial autocorrelation of the population death rate (Death_rate) in 108 prefecture-level cities in the YREB from 2010 to 2022. Table 2 reports the Global Moran's I and its statistical test results for the public health levels in the YREB.

**Table 2:** Global Moran's I of public health level.

Year	Moran's I	Z-score	*p-value*
2010	0.284^***^	3.452	0.001
2011	0.291^***^	3.518	0
2012	0.305^***^	3.689	0
2013	0.312^***^	3.745	0
2014	0.298^***^	3.612	0
2015	0.275^***^	3.385	0.001
2016	0.235^***^	2.912	0.004
2017	0.262^***^	3.214	0.001
2018	0.248^***^	3.056	0.002
2019	0.221^***^	2.785	0.005
2020	0.205^***^	2.642	0.008
2021	0.198^***^	2.558	0.011
2022	0.186^***^	2.415	0.016

^***^, ^**^, and ^*^, respectively indicate significance at the 1%, 5%, and 10% levels.

From the calculation results in Table 2, it can be seen that the Global Moran's I of the public health levels of cities in the YREB from 2010 to 2022 are all positive values, and the *p-values* of all years are less than 0.05 (most years less than 0.01), with Z scores greater than the critical value of 1.96. This indicates that during the entire study period, the death rates of 108 cities show a significant positive spatial autocorrelation. In other words, the public health levels are not randomly distributed in space but show obvious clustering characteristics: “high death rate” cities tend to be adjacent to “high death rate” cities (High-High clustering), while “low death rate” cities also tend to be adjacent to “low death rate” cities (Low-Low clustering). It is worth noting that observing the time-series changes from 2010 to 2022, the Global Moran's I is not constant but shows an inverted U-shaped fluctuation trend, with the overall intensity weakening. The first stage (2010–2013): the period of strengthening spatial agglomeration. The Moran's I index increased from 0.284 in 2010 to a peak of 0.312 in 2013. During this period, China was in the mid-stage of rapid industrialization and urbanization, and regional development imbalances intensified. The downstream areas took the lead in achieving rapid health level improvements through industrial upgrading and environmental governance, while the midstream and upstream areas were still trapped in environmental degradation brought by extensive growth, leading to a widening gap in health between regions. The spatial differentiation characteristics became more significant, and the Matthew effect of “the rich getting richer and the poor getting poorer” emerged in the health field, thereby increasing the degree of spatial autocorrelation. The second stage (2014–2022): the period of weakening spatial agglomeration. Since 2014, the Moran's I index has begun to show a fluctuating downward trend, gradually decreasing from 0.298 to 0.186 in 2022. Although it still maintains a significant positive correlation, the intensity of agglomeration has significant weakened. This may be due to the “YREB Development” rising to the level of a national strategy, especially the in-depth implementation of the concept of “jointly grasping great protection and not carrying out large-scale development.” The midstream and upstream areas have increased their efforts in environmental governance and medical infrastructure investment. The advancement of the equalization of basic public health services has quickly made up for the health shortcomings of backward areas, narrowing the absolute gap in health levels between regions. In addition, the improvement of the high-speed rail network and the popularization of internet healthcare have accelerated the penetration of high-quality medical resources to the central and western regions, weakening the limitations of geographical distance on health resource access, making the “health highland” effect of originally isolated areas diffuse outward, alleviating extreme spatial agglomeration.

### 5.4 SDM regression analysis

#### 5.4.1 Model selection and validation

To ensure the rigor of the model specification, this study adheres to a “general-to-specific” modeling logic, commencing with a rigorous statistical validation of the functional form of the baseline regression model. Based on the residuals derived from OLS regression, a Lagrange Multiplier (LM) test was conducted to ascertain the specific nature of spatial dependence present in the data (Table 3). The test results indicate that both the LM-lag statistic (44.98, *p* < 0.01) and the LM-error statistic (35.12, *p* < 0.01) are highly significant at the 1% significance level; furthermore, their corresponding robust forms (Robust LM-lag and Robust LM-error) also passed the significance tests. These findings demonstrate the presence of significant spatial autocorrelation within the data, suggesting that a SAR model or SEM may be insufficient to fully capture these complex spillover effects, thereby providing preliminary justification for the adoption of the more generalized SDM. Moreover, to further validate the appropriateness of the SDM, a Hausman test was performed to determine the appropriate form of effects; the results—with a statistic significant at the 5% level (*p* = 0.002)—led to the rejection of the null hypothesis of random effects, thereby confirming the applicability of a two-way fixed-effects model. In summary, grounded in the aforementioned rigorous statistical inferences, this study ultimately establishes the two-way fixed-effects SDM as the baseline regression model, thereby ensuring the robustness and reliability of the empirical analysis results.

**Table 3:** Model selection test results.

Test type	Statistic	*p*
LM-lag	44.98^***^	0.000
LM-error	35.12^***^	0.000
Robust LM-lag	15.45^***^	0.001
Robust LM-error	5.59^**^	0.018
Hausman test	28.45^***^	0.002
Wald test (SAR)	20.53^***^	0.000
Wald test (SEM)	18.12^***^	0.005
LR test (SAR)	19.88^***^	0.001
LR test (SEM)	17.56^***^	0.008

^***^, ^**^, and ^*^, respectively indicate significance at the 1%, 5%, and 10% levels.

#### 5.4.2 Regression results

The preceding Global Moran's *I* analysis has confirmed a significant positive spatial correlation in public health levels across cities in the YREB. This implies that OLS models may lead to estimation bias due to the neglect of spatial dependence. To accurately identify both the local effects and spatial spillover effects of urbanization on public health, this study further constructs a SDM. The SDM not only incorporates the spatial lag of the dependent variable (*W*×*Death*_*r*_*ate*) but also includes the spatial lags of the core explanatory variables and control variables. This specification effectively captures both the spatial spillovers of the dependent variable and the spatial interactions of the independent variables, making it the optimal model form for addressing such spatial dependencies. This paper selects the SDM as the baseline analytical framework and employs Maximum Likelihood Estimation (MLE) under fixed effects for parameter estimation.

The Table 4 shows that the spatial autoregressive coefficient ρ = 0.345 and is significantly positive at the 1% level. This key parameter indicates a strong positive spatial dependence in public health levels across the region: for every one-unit increase in the death rate of neighboring cities, the local city's death rate increases by an average of 0.345 units. This validates the conclusion drawn from the Moran's *I* test, suggesting that health risks (such as infectious diseases or pollution-induced illnesses) exhibit significant cross-regional transmission characteristics. Furthermore, competitive medical resource allocation or population mobility between adjacent areas intensifies this interconnected effect.

**Table 4:** The SDM regression results.

Variables	Coeff.	RobustSE	*Z*
*Urban*	−0.019^***^	(0.002)	−9.50
*W*×*Urban*	−0.012^***^	(0.003)	−4.00
*lnPGDP*	−0.008^***^	(0.002)	−4.00
*Med*	−0.001^*^	(0.001)	−1.75
*Pollution*	0.011^***^	(0.003)	3.67
*Green*	−0.003^**^	(0.001)	−2.15
*Aging*	0.025^***^	(0.003)	8.33
*W*×*lnPGDP*	−0.005^**^	(0.002)	−2.50
*W*×*Med*	0.001	(0.001)	1.10
*W*×*Pollution*	0.009^***^	(0.003)	3.00
*W*×*Green*	−0.002	(0.001)	−1.45
*W*×*Aging*	0.018^***^	(0.003)	6.00
ρ	0.345^***^	(0.045)	7.67
*_cons*	0.452^***^	(0.120)	3.77
*City/Year Fe*	Yes	-	-
*Log-Likelihood*	−1,856.24	-	-
*R* ^2^	0.782	-	-
*N*	1,404	-	-

The parentheses indicate robust standard errors; ^***^, ^**^, and ^*^, respectively indicate significance at the 1%, 5%, and 10% levels.

The coefficient for the urbanization rate is 0.019, significant at the 1% level. Although the absolute value is slightly smaller than that of the OLS model (0.024), it remains significantly negative, confirming the robustness of urbanization's ameliorative effect on local public health. The coefficient for the spatial lag of urbanization is 0.012, also significantly negative at the 1% level. This indicates that a region's urbanization process not only benefits local residents but also generates a significant positive spillover effect on neighboring cities (i.e., reducing their death rates). This “altruistic” effect may stem from: (1) the radiation of high-quality medical resources from central cities and service sharing; (2) regional transport integration accelerating emergency response and patient transfer; and (3) the diffusion of advanced environmental governance technologies and health concepts from urbanized areas to their peripheries. Among the spatial lags of control variables, the coefficient for *W*×*Pollution* is significantly positive (0.009), indicating that industrial pollution in neighboring areas significantly elevates local mortality, corroborating the transboundary transmission characteristics of air and water pollution. Meanwhile, *W*×*Aging* is significantly positive, reflecting the synchronicity of aging trends across regions and their collective impact on health system pressures.

#### 5.4.2 Effect decomposition

Because the SDM includes spatial lag terms, the regression coefficients of explanatory variables do not directly represent their marginal effects. As noted by Anselin and Elhorst, the total effect must be decomposed into direct, indirect, and total effects using partial differential methods (14, 15).

Table 5 shows that the direct effect of the urbanization rate is 0.021 (significant at 1%), implying that a 1 percentage point increase in local urbanization directly reduces the local death rate by 0.021 per thousand. This value is slightly higher than the regression coefficient in Table 3 because the direct effect encompasses feedback loops (i.e., local changes affect neighbors, which in turn affect the local area). More importantly, the indirect effect of urbanization (i.e., the spatial spillover effect) is 0.015 (significant at 1%). This suggests that an increase in local urbanization reduces the death rates of neighboring cities by 0.015 per thousand on average. In aggregate, the total effect of urbanization reaches 0.036, of which approximately 41.7% of the health dividend is realized through spatial spillover channels. This demonstrates that advancing new urbanization centered on city clusters can generate a “rising tide lifts all boats” regional health synergy.

**Table 5:** Effect decomposition results of SDM.

Variables	Direct effect	Indirect effect	Total effect
*Urban*	−0.021^***^	−0.015^***^	−0.036^***^
	(0.002)	(0.004)	(0.005)
*lnPGDP*	−0.009^***^	−0.006^**^	−0.015^***^
	(0.002)	(0.003)	(0.004)
*Med*	−0.001^*^	0.001	0.000
	(0.001)	(0.001)	(0.001)
*Pollution*	0.012^***^	0.011^***^	0.023^***^
	(0.003)	(0.004)	(0.005)
*Green*	−0.003^**^	−0.003^*^	−0.006^**^
	(0.001)	(0.002)	(0.002)
*Aging*	0.026^***^	0.022^***^	0.048^***^
	(0.003)	(0.004)	(0.005)

The parentheses indicate robust standard errors; ^***^, ^**^, and ^*^, respectively indicate significance at the 1%, 5%, and 10% levels.

The spatial externalities of other variables indicate that the indirect effect of environmental pollution is 0.011 and significantly positive, suggesting that local pollution not only harms local health but also severely endangers neighboring areas through air and water flow, with spillover damage nearly equivalent to local direct harm (direct effect 0.012). Conversely, per capita green space exhibits a significant negative spillover effect (0.003), indicating that urban ecological construction has positive externalities, as local increases in green space help improve the regional microclimate and benefit surrounding areas.

### 5.5 Quantile regression analysis

The OLS and SDM models discussed previously primarily focus on the impact of explanatory variables on the conditional mean of the dependent variable, revealing the “average effect” of urbanization on public health. However, mean regression often conceals heterogeneity in relationships across different distributional positions. In reality, cities with low public health levels (high mortality) and those with high public health levels (low mortality) may face significantly different constraints, resource endowments, and sensitivities to external shocks. For instance, in cities with poor health conditions, the marginal improvement effect of medical resources may be more pronounced (“charity in a snowstorm”); conversely, in cities with good health conditions, further urbanization may face diminishing marginal returns or constraints from “urban diseases.”

To comprehensively examine the distributional characteristics of urbanization's impact on public health and identify differences in its effects across health levels, this paper adopts Quantile Regression. We select five representative quantiles (τ = 0.10, 0.25, 0.50, 0.75, 0.90), corresponding to city samples with poor (top 10% mortality), fair (25%), moderate (50%), good (75%), and excellent (bottom 10% mortality) public health levels for estimation.

Table 6 demonstrates that across all selected quantiles, the coefficient for urbanization rate remains negative and predominantly statistically significant. This finding confirms that urbanization exerts a mortality-reducing effect irrespective of baseline public health conditions, thereby validating the robustness of our baseline regression results. Notably, however, the absolute value of the coefficient exhibits a distinct monotonic decreasing pattern, revealing both the asymmetric distribution of urbanization's health dividends and the phenomenon of diminishing marginal returns.

**Table 6:** Quantile regression results.

Variables	τ = 0.10	τ = 0.25	τ = 0.50	τ = 0.75	τ = 0.90	OLS
*Urban*	−0.035^***^	−0.029^***^	−0.024^***^	−0.018^***^	−0.012^**^	−0.024^***^
	(0.004)	(0.003)	(0.002)	(0.003)	(0.005)	(0.002)
*lnPGDP*	−0.018^***^	−0.014^***^	−0.010^***^	−0.008^**^	−0.006^*^	−0.010^***^
	(0.003)	(0.002)	(0.002)	(0.003)	(0.003)	(0.002)
*Med*	−0.005^***^	−0.003^**^	−0.002^*^	−0.001	−0.001	−0.002^*^
	(0.002)	(0.001)	(0.001)	(0.001)	(0.001)	(0.001)
*Pollution*	0.022^***^	0.018^***^	0.015^***^	0.011^**^	0.009^*^	0.015^***^
	(0.004)	(0.003)	(0.003)	(0.004)	(0.005)	(0.003)
*Green*	−0.006^***^	−0.005^**^	−0.004^**^	−0.003^*^	−0.002	−0.004^**^
	(0.002)	(0.002)	(0.002)	(0.002)	(0.002)	(0.002)
*Aging*	0.032^***^	0.030^***^	0.028^***^	0.025^***^	0.021^***^	0.028^***^
	(0.004)	(0.003)	(0.003)	(0.004)	(0.005)	(0.003)
*_cons*	8.120^***^	7.950^***^	7.680^***^	7.420^***^	7.150^***^	7.685^***^
*N*	1,404	1,404	1,404	1,404	1,404	1,404
*R* ^2^	0.685	0.712	0.748	0.725	0.698	0.754

The parentheses indicate robust standard errors; ^***^, ^**^, and ^*^, respectively indicate significance at the 1%, 5%, and 10% levels.

In the high-mortality quantile (τ = 0.10), representing cities with the most severe public health challenges (typically underdeveloped regions or legacy industrial bases in Central and Western China), the urbanization coefficient reaches its maximum absolute value of −0.035. This implies that for cities characterized by weak public health infrastructure and elevated mortality rates, a one-percentage-point increase in urbanization reduces the death rate by 0.035 per thousand—a magnitude substantially exceeding the average effect estimated by OLS (−0.024). Conversely, in the low-mortality quantile (τ = 0.90), comprising cities with the most advanced public health systems (primarily developed metropolitan areas), the urbanization coefficient diminishes to −0.012, with statistical significance attenuating to the 5% level. This attenuation indicates that in highly urbanized cities where health standards are already elevated, the marginal efficacy of additional urbanization in further reducing mortality becomes markedly constrained. For intermediate quantiles (τ = 0.25, 0.50, 0.75), the absolute value of the urbanization coefficient transitions smoothly from −0.029 to −0.018 as the quantile increases, delineating a clear linear decay trajectory. This pattern suggests that urbanization's health-promoting effects progressively weaken as the baseline health status improves.

Other control variables similarly exhibit pronounced quantile-dependent heterogeneity. The mortality-increasing effect of environmental pollution peaks in the high-mortality group (τ = 0.10, coefficient = 0.022) and reaches its nadir in the low-mortality group (coefficient = 0.009). This gradient implies that cities with inferior health conditions demonstrate heightened vulnerability to environmental shocks, wherein ecological degradation inflicts disproportionately severe damage on public health systems. In contrast, cities with robust health foundations, bolstered by superior medical defense capabilities and greater environmental governance investments, possess enhanced buffering capacity against environmental risks. The mortality-reducing effect of medical resources displays a systematic gradient across health status levels: it proves statistically significant in low-health groups (coefficient = −0.005) but becomes negligible in high-health groups. This finding reinforces the critical role of medical resources as “emergency relief” mechanisms and “safety nets”; for populations already enjoying optimal health, the marginal health returns from incremental increases in hospital bed capacity remain limited. Although aging exhibits significantly positive coefficients across all quantiles, its magnitude is notably larger in the high-mortality group (coefficient = 0.032), indicating that demographic aging imposes disproportionately greater strain on already fragile public health systems.

### 5.6 Endogeneity treatment: instrumental variable analysis

#### 5.6.1 Variable selection and construction

To address potential endogeneity issues between urbanization and public health, such as omitted variables and bidirectional causality, this study selects “terrain relief degree” (RDLS) as an instrumental variable. Terrain relief degree is a comprehensive metric calculated based on a Digital Elevation Model (DEM); typically defined as a function of the average elevation and relative height difference within a study area, it serves as a more scientifically rigorous indicator of the extent to which geomorphological features constrain human activities. Topography acts as a rigid constraint that fundamentally determines urban spatial morphology and the costs associated with urban expansion. Spanning China's three major topographic steps, the Yangtze River Economic Belt exhibits immense topographical diversity. The downstream Yangtze River Delta region is predominantly flat with an extremely low degree of terrain relief, facilitating urbanization primarily through horizontal expansion; conversely, the middle and upper reaches (such as the Yunnan-Guizhou Plateau and the fringes of the Sichuan Basin) feature fragmented terrain and high relief, where urbanization is often confined to river valleys and flat basins, manifesting as a “point-like” or “clustered” pattern of constrained development. Consequently, a naturally strong correlation exists between terrain relief degree and the rate of urbanization.

Furthermore, terrain relief degree is the product of the interplay between endogenous and exogenous geological forces acting over historical epochs, thereby falling under the category of natural endowments. It exists independently of contemporary socioeconomic systems, public health policies, and residents' lifestyles. Barring extreme geological disasters (such as earthquakes or landslides), terrain relief degree remains constant over the short term; thus, it exerts no direct influence on population mortality rates during the period, thereby satisfying the exclusion restriction required of an instrumental variable.

#### 5.6.2 Two-stage least squares (2SLS) estimation

Based on the aforementioned instrumental variables, this paper employs the 2SLS method for estimation, in order to simultaneously address endogeneity and spatial dependence. As shown in Table 7, the results from the first stage indicate that the coefficient for RDLS is −0.152, and it is highly statistically significant at the 1% level. This provides strong evidence that regions with more rugged terrain exhibit significantly lower urbanization rates—a finding consistent with the “geographic resistance” hypothesis and satisfying the relevance requirement for instrumental variables. The F-statistic is 48.25, which is substantially greater than the critical value for the Stock-Yogo test (typically 10), indicating that the instrumental variable selected in this study is not a “weak instrument.”

**Table 7:** Instrumental regression results.

Variables	First stage	Second stage
*Urban* *(endogenous variable)*	*Death*_*rate* (explained variable)	
*I* *V(RDLS)*	−0.152^***^ (0.035)	-
*Urban*	-	−0.029^***^ (0.004)
*Control variable*	Yes	Yes
*Spatial lag term (W*×*X)*	Yes	Yes
*City/Year Fe*	Yes	Yes
*Cragg-Donald Wald F*	48.25 (>10)	-
*N*	1,404	1,404

The parentheses indicate robust standard errors; ^***^, ^**^, and ^*^ respectively indicate significance at the 1%, 5%, and 10% levels.

The results from the second-stage regression reveal that, after addressing the issue of endogeneity, the coefficient for the Urbanization Rate is −0.029, and it is significantly negative at the 1% level. The absolute value of this coefficient is slightly larger than that obtained in the baseline regression; this suggests that when endogeneity is not taken into account, OLS or fixed-effects models may underestimate the positive impact of urbanization on public health (implying an upward bias). These empirical findings confirm that, even within the most rigorous framework for causal identification, urbanization remains a key driving force for reducing mortality rates and enhancing public health levels within the Yangtze River Economic Belt.

### 5.7 Mechanism testing: transmission path analysis

The baseline regression has confirmed that urbanization significantly improves public health. To delve deeper into the underlying mechanisms of this influence, this study constructs a three-dimensional transmission framework: “Medical Technology Upgrading—Resident Income Growth—Environmental Quality Improvement.” Notably, to avoid logical overlap and “over-control” bias with the “number of beds per thousand people” (a proxy for medical resource stock) used as a control variable in the baseline regression, this section selects an indicator that better represents medical technology quality and structural upgrading for the medical path: the “Proportion of Grade 3A Hospitals” (the ratio of Class 3 Grade A hospitals to the total number of hospitals in the region). While the bed count in the baseline regression mainly reflects the “scale” of medical services, the proportion of Grade 3A hospitals reflects the “capability level” and technological intensity of medical services. Urbanization not only brings scale expansion but also drives the structural upgrading of high-quality medical resources through agglomeration effects, which is the key mechanism for enhancing the treatment capacity for difficult and critical illnesses and reducing mortality. All models control for city and year fixed effects and incorporate spatial lag terms to eliminate spatial dependence interference.

Column (1) of Table 8 shows that the regression coefficient of urbanization on the proportion of Grade 3A hospitals is 0.038 and significant at the 1% level. This indicates that the urbanization process significantly promotes the agglomeration and structural upgrading of high-quality medical resources. As the population concentrates in cities, large cities, leveraging advantages in talent, research, and capital, attract more high-level hospitals or facilitate the upgrading of existing hospitals. In the second-stage regression, the coefficient of the Grade 3A hospital proportion is −0.115 and significantly negative, indicating that the enhancement of medical technology capability significantly reduces mortality. At this point, the coefficient of urbanization decreases from −0.019 in the baseline model to −0.016. The Sobel test *Z-value* is 4.12, with a mediation effect accounting for 24.53%, the largest among the three paths. This result reveals that the core of urbanization's health improvement lies not merely in “having a bed,” but in “having good doctors and good technology.” The agglomeration effect brought by urbanization allows difficult and complex diseases to be treated more efficiently locally, reducing deaths caused by treatment delays and validating the health dividend of “quality-oriented urbanization.”

**Table 8:** Results of mechanism testing.

Variables	(1) Medical technology path	(2) Income growth path	(3) Environmental governance path
In the first-stage			
*Explained variable*	*Tech*	ln*Income*	*SO*_2_
*Urban*	0.038^***^ (0.004)	0.041^***^ (0.003)	−0.035^***^ (0.005)
*Control variable*	Yes	Yes	Yes
*City/Year Fe*	Yes	Yes	Yes
In the second-stage			
*Explained variable*	*Death*_*rate*	*Death*_*rate*	*Death*_*rate*
*Urban*	−0.016^***^ (0.003)	−0.015^***^ (0.003)	−0.014^***^ (0.003)
*Tech*/*lnIncome*/*SO*_2_	−0.115^***^ (0.015)	−0.068^***^ (0.009)	0.052^***^ (0.010)
*Sobel Z*	4.12^***^	3.85^***^	3.21^***^
*Proportion of intermediary effect*	24.53%	22.18%	18.65%

The parentheses indicate robust standard errors; ^***^, ^**^, and ^*^ respectively indicate significance at the 1%, 5%, and 10% levels.

Column (2) of Table 8 shows that urbanization significantly increases per capita disposable income of urban residents (coefficient 0.041), and income growth significantly suppresses mortality (coefficient −0.068). After including the income variable, the urbanization coefficient decreases to −0.015, with a mediation effect accounting for 22.18%. Unlike macro-level per capita GDP, disposable income directly determines household consumption budgets. Urbanization, by providing more high-value-added jobs, practically increases residents' income, enabling households to afford more balanced nutrition, fitness activities, commercial health insurance, and preventive medical check-ups. This enhancement in “health investment capacity” at the micro-level constitutes an important micro-foundation for translating urbanization into health outcomes.

Column (3) of Table 8 shows that the coefficient of urbanization on SO_2_ emission intensity is −0.035, indicating that urbanization significantly reduces pollution emissions per unit of economic output. Meanwhile, the impact coefficient of SO_2_ emission intensity on mortality is 0.052 (significantly positive). After including the environmental variable, the urbanization coefficient decreases to −0.014, with a mediation effect accounting for 18.65%. This provides strong support for the applicability of the “Environmental Kuznets Curve” hypothesis in the YREB. Urbanization does not inevitably lead to environmental deterioration. On the contrary, high-level urbanization is accompanied by industrial structure upgrading (“moving from secondary to tertiary industries”—high-pollution industries relocating or transforming, with a rising share of services) and stricter enforcement of environmental regulations. This “structural emission reduction” and “technological emission reduction” driven by urbanization effectively improve regional air quality, thereby reducing mortality from respiratory diseases.

### 5.8 Robustness test

The baseline regressions, endogeneity treatments, and mechanism tests presented above consistently indicate that the urbanization process has significantly reduced population mortality rates in cities along the YREB, demonstrating causal reliability. However, the robustness of empirical results may be affected by the singularity of core variable measurements. Different index construction methods, data sources, or statistical criteria may lead to estimation biases. To eliminate the risk of “spurious regression” caused by accidental variable selection and ensure the universality and robustness of the research conclusions, this section conducts robustness checks using two strategies: replacing core explanatory variables and replacing the dependent variable.

#### 5.8.1 Variable replacement

##### 5.8.1.1 Replacement of the core explanatory variable: from “Population Urbanization” to “Land Urbanization”

In the baseline regression, the core explanatory variable was the permanent population urbanization rate, i.e., the proportion of the permanent urban population to the total population. Although this indicator can reflect the degree of population agglomeration, under China's specific institutional context, there may be a “semi-urbanization” phenomenon (i.e., the population is statistically classified as urban but does not fully enjoy citizen benefits). Furthermore, China's urbanization is often accompanied by large-scale land expansion. Therefore, this study selects “built-up area proportion” as an alternative indicator, i.e., the proportion of the urban built-up area to the total administrative area. This indicator measures the level of urbanization from the perspective of spatial form and land development intensity, capturing the potential impact of infrastructure expansion and physical space restructuring on public health. If the regression results based on this indicator remain significant, it indicates that the health-improving effect of urbanization stems not only from population agglomeration but also from the optimization of the urban spatial carrier.

##### 5.8.1.2 Replacement of the dependent variable: from “Crude Death Rate” to “Standardized Death Rate”

The dependent variable in the baseline regression was the population crude death rate. Although the crude death rate is intuitive, its value is highly susceptible to the influence of regional population age structure. There are significant differences in the degree of aging among cities within the YREB (e.g., deeply aging cities such as Shanghai and Nantong coexist with young cities in the west). If the interference of age structure is not completely eliminated, the true health effect of urbanization may be overestimated or underestimated. To this end, this study adopts the “age-standardized death rate” as an alternative dependent variable. This indicator uses the age-specific population structure of the 2010 Sixth National Population Census as the standard population to weight the age-specific death rates of each city, thereby eliminating the incomparability caused by age structure differences between cities and more purely reflecting the impact of medical and health levels and living environments on death risk.

#### 5.8.2 Robustness analysis

Based on the above-replaced variables, this study re-ran the SDM, keeping the control variables, fixed effects, and spatial weight matrix consistent with the previous text.

Column (1) of Table 9 shows the regression results using “built-up area proportion” to replace “population urbanization rate.” The results indicate that the direct effect coefficient of Land_Urban is −0.021 and highly significant at the 1% level; the indirect effect (spatial spillover) coefficient is −0.018, also significantly negative. Specifically, regardless of whether the population or land indicator is used, the effect of urbanization on reducing the death rate is significantly negative, confirming that the baseline conclusion is not dependent on the measurement bias of a single indicator. The total effect of land urbanization (−0.039) is slightly higher than that of population urbanization, suggesting that in the Yangtze River Economic Belt, the marginal contribution of the expansion of urban physical space (e.g., new district construction, infrastructure improvement, green space increase) to public health should not be overlooked.

**Table 9:** Robustness test.

Variables	(1) Replace explanatory variables	(2) Replace the explained variable
Direct effect		
*Land_Urban/* *Urban*	−0.021^***^ (0.004)	−0.017^***^ (0.003)
*t*	−5.25	−5.67
Indirect/Spillover effect		
*Land_Urban/* *Urban*	−0.018^***^ (0.005)	−0.014^***^ (0.004)
*t*	−3.60	−3.50
Total effect		
*Land_Urban/* *Urban*	−0.039^***^ (0.006)	−0.031^***^ (0.005)
*Control variable*	Yes	Yes
ρ	0.285^***^	0.262^***^
*City/Year Fe*	Yes	Yes
*N*	1,404	1,404
*R* ^2^	0.742	0.768

The parentheses indicate robust standard errors; ^***^, ^**^, and ^*^ respectively indicate significance at the 1%, 5%, and 10% levels.

Column (2) shows the regression results using the “age-standardized death rate” as the dependent variable. After eliminating the interference of population age structure, the direct effect coefficient of Urban is −0.017, and the indirect effect is −0.014, both significantly at the 1% level. Although some developed cities in the YREB have high crude death rates due to severe aging, urbanization still shows a significant health-promoting effect after controlling for age structure. This indicates that the positive forces brought by urbanization, such as medical technology progress, income increase, and environmental improvement, are sufficient to offset and even surpass the negative pressure brought by aging. The coefficient after standardization (−0.017) is very close in magnitude to the crude death rate coefficient in the baseline regression (−0.019), only slightly reduced. This suggests that although age standardization was not performed in the baseline regression, the bias of age structure was to some extent alleviated because the “aging rate” variable had already been controlled in the model, confirming the reliability of the baseline results.

## 6 Conclusion and policy implications

### 6.1 Research conclusions

Based on panel data from 108 prefecture-level cities in the YREB from 2010 to 2022, this study systematically examined the impact, spatial spillover characteristics, and internal transmission mechanisms of the urbanization process on public health levels using a SDM, quantile regression, and causal mediation effect analysis. The main conclusions are as follows:

First, urbanization has significantly elevated regional public health levels, demonstrating a robust causal inhibitory effect on mortality. After controlling for economic development, environmental pollution, medical resource availability, and population structure, the data reveal that an increase in the urbanization rate drives improvements in public health. These findings strongly support the “urban bonus” hypothesis within the Yangtze River Economic Belt (YREB), indicating that the agglomeration economies, optimized public services, and lifestyle enhancements generated by urbanization collectively outweigh the negative externalities associated with “urban diseases.” Consequently, urbanization has emerged as a pivotal driver for reducing death risks and extending life expectancy.

Second, urbanization exerts significant positive spatial spillover effects on public health. The urbanization process in a local city not only improves the health status of its own residents but also significantly reduces mortality rates in neighboring cities through mechanisms such as factor flows, technology diffusion, and joint environmental governance. Specifically, a 1% increase in the urbanization rate of neighboring cities corresponds to an additional 0.015% reduction in local mortality rates. Notably, these spatial spillover effects account for 41.7% of the total impact, underscoring the regional interdependence of health governance and confirming the health externalities inherent in coordinated urban agglomeration development.

Third, the health impacts of urbanization exhibit significant heterogeneity. Quantile regression results indicate that the marginal benefit of urbanization on public health is substantially stronger in high-mortality regions—primarily underdeveloped cities in the middle and upper reaches—compared to low-mortality regions, which are predominantly developed cities in the lower reaches. At the high-mortality quantile, the absolute value of the urbanization rate coefficient reaches −0.035%, whereas at the low-mortality quantile, it is merely −0.012%, suggesting that urbanization yields greater health returns where baseline health conditions are poorer.

Fourth, medical technology upgrading, resident income growth, and environmental governance effectiveness constitute three core transmission pathways. Mediation analysis quantifies their respective contributions: improvements in medical technology explain 24.53% of the total effect, highlighting the agglomeration of high-quality medical resources as the primary channel; resident income growth accounts for 22.18%, validating income as a critical input in the health production function; and environmental governance effectiveness contributes 18.65%, confirming the pivotal role of green transformation in mitigating the negative externalities of urbanization.

Although this study innovates in its theoretical framework and empirical methods, several limitations warrant further investigation. First, the measurement of public health relies primarily on the crude death rate due to macro-data constraints; future research should incorporate micro-survey data or multi-dimensional health indices for a more comprehensive assessment. Additionally, we explicitly acknowledge the limitation of using a single pollutant indicator; subsequent studies should integrate multi-pollutant indices (including PM2.5, NOx, etc.) to provide a holistic evaluation of environmental exposure. Second, while quantile regression reveals heterogeneity, the potential “inverted U-shaped” or threshold effects between urbanization and health remain underexplored. Future work is needed to identify optimal urbanization scales where the negative externalities of “urban diseases” begin to outweigh agglomeration benefits, thereby clarifying the precise boundaries between health promotion and deterioration.

### 6.2 Policy implications

Based on the significant causal effects, spatial spillover characteristics, and multi-dimensional transmission mechanisms of urbanization on public health revealed in this study, it is urgent to reconstruct the policy framework across three levels: differentiated strategic deployment, regional coordinated governance, and multi-dimensional path optimization. This reconstruction aims to maximize the health dividends of the YREB by leveraging existing spatial dynamics rather than prescribing specific site selections.

First, implement a differentiated urbanization health promotion strategy that aligns with regional heterogeneity. The quantile regression results confirm significant heterogeneity in the marginal improvement effect of urbanization on public health; health benefits in the middle and upper reaches (characterized by higher mortality) are distinct from those in the developed lower reaches. Instead of prescribing specific “priority locations” for new infrastructure, policy resources should be strategically tilted to support capacity building in underdeveloped regions. For node cities in the middle and upper reaches, the focus should be on orderly expanding city size and increasing urbanization rates within ecological carrying capacities, while optimizing the allocation efficiency of existing medical resources (32, 37, 38). Specifically, transfer payments for public health infrastructure should be increased to bridge resource gaps through agglomeration effects, ensuring that medical facilities are distributed where they can most effectively serve local populations. Conversely, for high-urbanization areas like the YREB, which have entered a post-industrial stage, the policy focus must shift from “scale expansion” to “connotation quality improvement.” The goal is to govern potential health risk factors associated with over-agglomeration—such as traffic congestion, housing costs, and social psychological pressure—to prevent the marginal decrease or reversal of health benefits.

Second, construct a cross-administrative boundary “Health Community” coordinated governance mechanism to fully release the spatial spillover effects of urbanization. The SDM results demonstrate that local urbanization generates significant positive health spillovers to neighboring cities, with a contribution rate reaching 41.7%. This indicates that health governance possesses strong externalities and regional relevance, rendering single-city “island-style” governance ineffective. Therefore, traditional administrative barriers must be broken to establish a paradigm of YREB-wide linkage health governance. Rather than focusing on the physical construction of specific centers in isolated locations, the priority should be on institutional synergy: upgrading the “Yangtze Health Community” mechanism based on existing integrated development frameworks (e.g., Yangtze River Delta integration and Chengdu-Chongqing twin-city economic circle). The objective is to create a coordinated pattern where urbanization in one area facilitates shared health outcomes across the region, ensuring that the benefits of medical technology and resource flow freely across administrative boundaries (17, 23, 39).

Finally, we advocate for deepening the three-dimensional linkage reform integrating medical technology, resident income, and environmental governance to precisely unblock and reinforce the transmission pathways identified in our analysis. The mediation effect analysis quantifies the distinct contributions of these three core mechanisms: medical technology upgrading (24.53%), resident income growth (22.18%), and environmental governance effectiveness (18.65%). Consequently, policy interventions should be strategically targeted at these specific channels to maximize their efficiency. In the realm of medical technology, priority should be given to “quality upgrading” and “spatial optimization” through mandating high-standard medical land reservations within urban planning frameworks. This approach must extend beyond new construction to actively facilitate the sinking and diffusion of high-quality medical resources from central hubs to peripheral areas, thereby amplifying technological spillover effects. Regarding resident income, accelerating industrial transformation toward high-value-added, low-pollution sectors is essential for generating quality employment opportunities. This strategy aims to steadily elevate resident disposable income, thereby fortifying the economic foundation essential for health production. With respect to environmental governance, strict adherence to ecological red lines should be coupled with green industrial transformation and energy structure optimization (27, 32, 40). By enhancing urban green space systems, ventilation corridors, and blue-green ecological networks, policymakers can effectively integrate the “green content” of ecological spaces into the “health content” of residents' lives, thereby mitigating the negative externalities associated with rapid urbanization (35, 36, 41).

## Data Availability

The original contributions presented in the study are included in the article/supplementary material, further inquiries can be directed to the corresponding author.
